# Tilden and Co’s Medicinal Extracts

**Published:** 1850-01

**Authors:** 


*Tilden and Co's Medicinal extracts.*—A few specimens of the medicinal extracts prepared by Messrs Tilden and Co. of New York, have been sent us for examination, with the appearance of which we are much pleased. We are informed that the mode of preparation of these articles, by this Co., is different from that usually employed, the evaporation being conducted rapidly, and at a very low temperature. The active properties of the substances treated in this way are preserved unaltered. The extracts are put up in bottles very securely protected from the atmosphere, and are contained in handsome pasteboard boxes, presenting a neat and even elegant appearence. We have not, as yet, had time to test their comparative efficacy, but we are assured, upon the authority of those who have done so, that they may be depended upon as representing faithfully the medicinal virtues of the plants from which they are derived. Every practitioner who has been in the habit of prescribing medicinal extracts, must have had abundant reasons to know that articles, domestic and foreign, in common use, are frequently nearly worthless. So often have we found this to be the case, that we have, on that account, on many occasions refrained from prescribing them in cases in which they have been indicated. The frequency of complaints of this kind has induced Messrs. Tilden & Co. to devote to their preparation especial care, and to offer them in a form in which their house may be held responsible for the excellence of the articles. The plan appears to us calculated to do away, in some measure, at least, with adulterated and inert domestic medicines.

With respect to the extracts above referred to, we copy the following letter by Prof. Clark of the city of New York, which we find in the New York Journal of Medicine No. for Nov.

"I have lately visited the manufactory of these extracts. After inspecting the whole process and examining a large number of preparations, I became so fully persuaded that these gentlemen have fallen upon the best plan of concentrating and preserving the active principles, especially of the narcotic vegetables, that I have voluntarily offered to them any assistance that I can render in introducing their medicines to the notice of the professton; being persuaded that these extracts must posess the efficiency and the uniformity of strength so necessary to the successful use of this class of remedies, and I may add, so long sought for in vain.

Should your conviction of the value of these preparations correspond with my own, after you have examined them and tried them in practice, perhaps you may think it due alike to the profession, and to the gentlemen who are improving the instruments by which we work. to call the attenrion of our readers to the improvements which I cannot doubt this process secures."

